# Lights and Shadows of Clinical Applications of Cardiac Scintigraphy with Bone Tracers in Suspected Amyloidosis

**DOI:** 10.3390/jcm12247605

**Published:** 2023-12-10

**Authors:** Riccardo Saro, Daniela Pavan, Aldostefano Porcari, Gianfranco Sinagra, Marco Mojoli

**Affiliations:** 1 Center for Diagnosis and Treatment of Cardiomyopathies, Cardiovascular Department, Azienda Sanitaria Universitaria Giuliano-Isontina (ASUGI), University of Trieste, Via P. Valdoni 7, 34100 Trieste, Italy; riccardo.saro@gmail.com (R.S.); aldostefanoporcari@gmail.com (A.P.); gianfranco.sinagra@asugi.sanita.fvg.it (G.S.); 2 European Reference Network for Rare, Low Prevalence and Complex Diseases of the Heart-ERN GUARD-Heart, Giuliano Isontina University Health Authority, 34149 Trieste, Italy; 3 Ospedale Santa Maria degli Angeli, Azienda Ospedaliera Friuli Occidentale (ASFO), 33170 Pordenone, Italy; daniela.pavan@asfo.sanita.fvg.it; 4 National Amyloidosis Centre, Division of Medicine, University College London, Royal Free Campus, Rowland Hill Street, London NW3 2PF, UK

**Keywords:** cardiac amyloidosis, cardiomyopathies, HFpEF, bone scintigraphy, bone tracers, multidisciplinary teams

## Abstract

Radionuclide bone scintigraphy is the cornerstone of an imaging-based algorithm for accurate non-invasive diagnosis of transthyretin cardiac amyloidosis (ATTR-CA). In patients with heart failure and suggestive echocardiographic and/or cardiac magnetic resonance imaging findings, the positive predictive value of Perugini grade 2 or 3 myocardial uptake on a radionuclide bone scan approaches 100% for the diagnosis of ATTR-CA as long as there is no biochemical evidence of a clonal dyscrasia. The technetium-labelled tracers that are currently validated for non-invasive diagnosis of ATTR-CA include pyrophosphate (^99m^Tc-PYP); hydroxymethylene diphosphonate (^99m^Tc-HMDP); and 3,3-diphosphono-1,2-propanodicarboxylate (^99m^Tc-DPD). Although nuclear scintigraphy has transformed the contemporary diagnostic approach to ATTR-CA, a number of grey areas remains, including the mechanism for binding tracers to the infiltrated heart, differences in the kinetics and distribution of these radiotracers, differences in protocols of image acquisition worldwide, the clinical significance of extra-cardiac uptake, and the use of this technique for prognostic stratification, monitoring disease progression and assessing the response to disease-modifying treatments. This review will deal with the most relevant unmet needs and clinical questions concerning scintigraphy with bone tracers in ATTR-CA, providing expert opinions on possible future developments in the clinical application of these radiotracers in order to offer practical information for the interpretation of nuclear images by physicians involved in the care of patients with this ATTR-CA.

## 1. Introduction

Amyloidosis is a disease that remains largely unexplored with several unsolved challenges and encompasses a heterogeneous group of diseases, acquired or inherited, that may occur in a systemic or localized form and share a pathogenetic mechanism of amyloid fibril deposition in the extracellular space of various organs [1]. Heart involvement, namely cardiac amyloidosis (CA), is the main determinant of long-term outcome [2].

Although there are more than 30 different precursor proteins (the number appears to be significantly higher based on mass spectrophotometry), the most frequent forms of CA are caused by transthyretin (ATTR) and immunoglobulin light chain (AL) precursor proteins [3]. ATTR amyloidosis includes a non-hereditary form (ATTRwt) and a hereditary form due to mutations in the transthyretin gene called variant amyloidosis (ATTRv) [2]. The disease occurs when the structural integrity and consequently the function of the tissues affected by the deposition of amyloid fibrils is lost. ATTRwt amyloidosis exhibits greater involvement of the heart, while ATTRv amyloidosis leads to both cardiomyopathy and polyneuropathy [4].

The epidemiology of amyloidosis, once considered a rare and incurable disease, has been rewritten in recent years thanks to important innovations in the diagnostic and therapeutic fields [5]. The diagnosis of amyloidosis was once reached only by biopsy (heart, subcutaneous fat, salivary gland, or rectum biopsies) and usually at an advanced level of pathology [6] probably due to low awareness of the condition, frequent misdiagnosis, and the lack of a viable, non-invasive diagnostic approach. Currently, owing to advances in cardiac magnetic resonance imaging (MRI) and cardiac scintigraphy with bone tracers, cardiac transthyretin amyloidosis (ATTR-CA) can be diagnosed non-invasively and usually at an earlier stage of disease. Cardiac scintigraphy with bone tracers has brought a revolution in the non-invasive diagnosis of ATTR-CA mainly due to the contribution of the algorithm proposed by Gillmore et al. [7], where scintigraphy achieved excellent sensitivity and specificity values (99% and 86%, respectively) and a positive predictive value of 100% when combined with serum and urinary immunofixation and negative immunoglobulin light chain assay results for the monoclonal component. 

However, despite the enormous progress in the non-invasive diagnosis of ATTR-CA in recent years, cardiac scintigraphy with bone tracers still presents several mysteries. The mechanism by which bone tracers accumulate in ATTR-CA patients is yet unknown, and possible further applications of cardiac scintigraphy beyond the diagnosis of amyloidosis remain undiscovered.

This review addresses this by presenting what we know to date and provides insights into future perspectives in the field of cardiac scintigraphy with bone tracers.

## 2. Bone Tracers and Cardiac Amyloidosis

### 2.1. Pathophysiological Mechanisms of Amyloidogenic Cascade

Amyloidosis is a systemic disease characterized by the deposition of type A or type B fibrillar amyloid proteins (7–13 nm in diameter) with a classic beta-strand hydrogen-bonded structure. Type A fibrils are composed of full-length and C-terminal fragments of transthyretin, while type B fibrils comprise only full-length transthyretin. Amyloid fibrils are insoluble polymers consisting of various protein subunits that are in turn formed from soluble precursors that, through conformational changes, obtain a beta-sheet configuration. Within these deposits, serum amyloid P components and glycosaminoglycans can be found [1]. At the beginning of fibrillogenesis and nucleated polymerization, several molecules, which may be unfolded, partially folded, or completely folded become nuclei and aggregate to generate totally disordered, partially disordered, or structured oligomers. These aggregates can then fragment and trigger second nucleation reactions, thus creating new nuclei. As the aggregation process proceeds, these oligomers may acquire a beta-sheet structure and then continue to grow by aggregating with each other or with other monomers. The process of fibrillogenesis is facilitated by partial folding or unfolding of the precursors, which is accelerated by acidification, proteolysis, primary nucleation, fibril fragmentation, and secondary nucleation.

At this point, the toxicity of amyloid fibrils is due to their deposition in the extracellular space of organs and tissues, undermining their structural integrity [8].

### 2.2. Cardiac Scintigraphy

The diagnosis of CA was previously established by endomyocardial biopsy (EMB), which was associated with procedural risks and the need to be performed in capable hands; the histological preparation that is stained with Congo red and placed under polarized light shows the pathognomonic apple-green birefringence. Bone tracer scintigraphy is a nuclear medicine diagnostic method that uses radiation emitted by a radiopharmaceutical labelled with radioactive isotopes previously injected intravenously and detected using a suitable instrument, the gamma-camera, allowing areas of tracer accumulation to be identified. Irradiation is minimal (average 3.2 mSv), and no side effects are described. Thus, this technique can be performed in anyone, regardless of renal function, with the exception of pregnant or breastfeeding women. Furthermore, contact with children must be avoided 24 h after exposure. Thanks to the contribution provided initially by Perugini and later by the protocol proposed by Gillmore et al. as well as the consolidation of their findings with the publication of the position statement of the European Society of Cardiology and the very recent guidelines on cardiomyopathies, myocardial scintigraphy with bone tracers allows the diagnosis of ATTR-CA without the need for EMB in selected cases [7]. 

### 2.3. Bone Tracers

Tracers used for bone scintigraphy are diphosphonates or pyrophosphates bound to metastable technetium-99 (99mTc), and their accumulation at the cardiac level has been described since the 1980s. However, they have been studied in a more comprehensive approach for cardiac amyloidosis from the 2000s onwards, especially since Perugini’s work [9,10].

**Figure 1:**
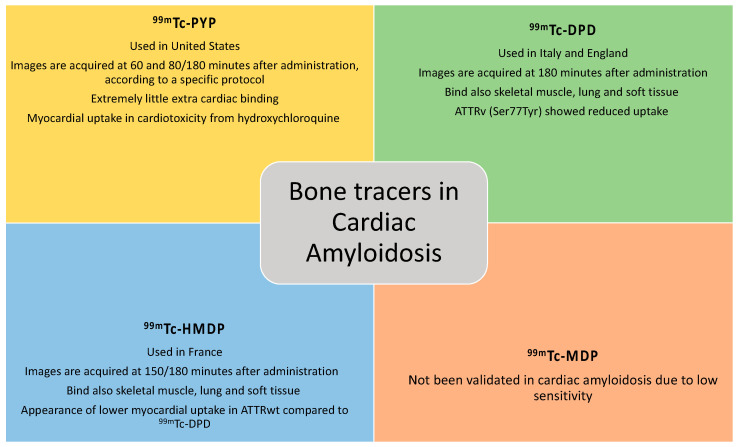
Bone tracers currently used to identify cardiac amyloid deposits. ^99m^Tc-PYP = Technetium-99m-pyrophosphate; ^99m^Tc-DPD = Technetium-99m-3,3-diphosphone-1,2-propan-dicarboxylic acid; ^99m^Tc-HMDP = Technetium-99m-hydroxymethylene-diphosphonate; ^99^mTc-MDP = Technetium-99m-methylene diphosphonate; ATTRv = amyloid transthyretin variant; ATTRwt = amyloid transthyretin wild-type.

Bone tracers currently used to identify cardiac amyloid deposits (Figure 1) include ^99m^Tc-pyrophosphate (^99m^Tc-PYP) [11], the only one employed in the United States; ^99m^Tc-3,3-diphosphone-1,2-propan-dicarboxylic acid (^99m^Tc-DPD) [12], employed in the United Kingdom and Italy (and generally in Europe); and ^99m^Tc-hydroxymethylene-diphosphonate (^99m^Tc-HMDP) [13], which is used in France. All of these tracers are essentially analogues from a diagnostic point of view [14]. Although other bone tracers exist, they are not validated for non-invasive confirmation of ATTR-CA. For example, ^99m^Tc methylene diphosphonate (^99^mTc-MDP) has been associated with low sensitivity towards detection of cardiac amyloid infiltration and false negative results. Post-injection, the ^99^mTc-PYP image is acquired at 60 min and 80 min or 3 h (based on local protocol), and that of ^99^mTc-HMDP is acquired at 2.5 or 3 h.

However, the mechanism by which these tracers bind amyloid and why uptake is greater in ATTR amyloidosis than in AL remain unknown. It has been suggested that this difference may be due to a greater presence of microcalcifications in ATTR amyloidosis than in AL [15,16] or to the different type of fibrils associated with the C-terminal fragments of the protein. For example, type A fibrils, which are those associated with the C-terminal fragments TTR, were able to bind ^99m^Tc-DPD, whereas type B did not [17]. Interestingly, in this recent and well conducted study [15], the authors analyzed three hearts from patients with ATTR amyloidosis and three hearts from patients with AL amyloidosis. On histology, the authors demonstrated the presence of microcalcifications in a dust-like form in all three ATTRwt hearts and in two out of three AL hearts, however, in much smaller amounts; this would explain why cardiac scintigraphy with bone tracers can show a certain degree of uptake even in patients with AL amyloidosis. Surprisingly, the authors pointed out that the presence of microcalcifications is not necessarily associated with cardiac amyloid deposits [15]. 

Compared to other bone tracers, ^99m^TC-PYP accumulates substantially in myocardial TTR deposits, while it exhibits extremely little extracardiac binding [18]. In contrast, with ^99m^Tc-DPD/HMDP, amyloid deposition was also revealed in skeletal muscle, lung and soft tissue [19]. 

There is only a single study designed as a head-to-head comparison of clinical accuracy of different bone tracers in patients with suspected CA and has been conducted at the National Amyloidosis Centre (NAC) in a cohort of patients being scanned with ^99m^Tc-HMDP (locally) and, later, with ^99m^Tc-DPD (at the NAC) [20]. The median time interval between the first and second radionuclide scan was less than 5 months. The Perugini grade differed between ^99m^Tc-HMDP and ^99m^Tc-DPD in 33% patients, all of whom had wild-type ATTR-CA, with lower myocardial uptake on ^99m^Tc-HMDP (grade 1) in all such cases compared with ^99m^Tc-DPD (grade 2). Based on these findings, a prospective head-to-head comparison of the three approved radiotracers is required in the near future.

### 2.4. Cardiac Evaluation Methods

**Figure 2:**
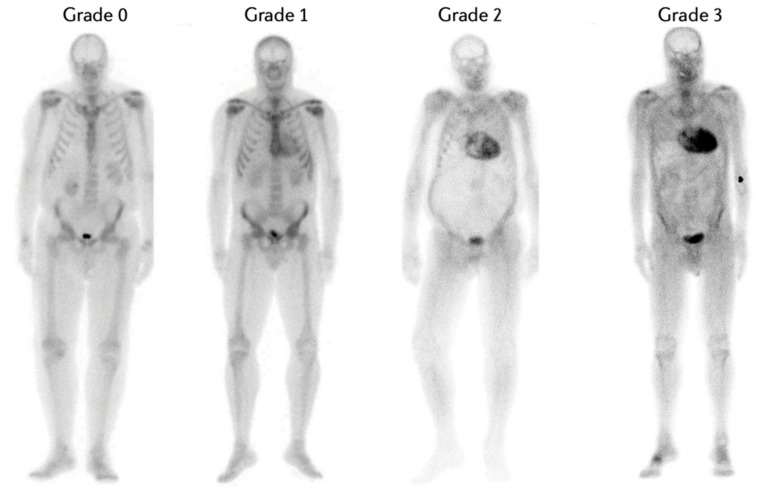
Degree of cardiac uptake on planar scintigraphy. This figure, which is public, was taken with the author’s consent from the article by Porcari et al. [4].

There are several quantitative and qualitative methods utilized to assess the presence and degree of bone tracer accumulation in the heart. The acquisition of both antero-posterior and latero-lateral projection is crucial to discriminate the site of accumulation on planar imaging. The most commonly used is the Perugini score, which qualitatively assesses the degree of cardiac uptake compared to bone uptake on planar images. Tracer uptake is then classified into 4 categories (Figure 2) [4]: grade 0, no cardiac uptake; grade 1, mild cardiac uptake that is less than bone uptake; grade 2, moderate cardiac uptake accompanied by attenuated bone uptake; and grade 3, strong cardiac uptake with mild/absent bone uptake [10,21]. Recently, a new modified Perugini score has been proposed by Dorbala et al., in which radiotracer uptake is compared with bone uptake and in particular with rib uptake. The score is classified as follows: grade 0, no myocardial uptake and normal bone uptake; grade 1, myocardial uptake less than rib uptake; grade 2, myocardial uptake equal to rib uptake; and grade 3, myocardial uptake greater than rib uptake with mild/absent rib uptake. However, clinical application of this modified score in the real world has not been validated to date [22].

Semi-quantitative methods to assess the degree of myocardial uptake of bone tracers include the heart to contralateral ratio (H/CL), heart to whole body ratio (H/WB), heart to pelvis ratio (H/P), and heart to skull ratio (H/S) (Table 1).

In particular, the H/CL method, validated only in scintigraphy with ^99m^Tc-PYP, involves comparing two regions of interest (ROI) on planar antero-posterior images. The first region is drawn around the heart avoiding the inclusion of the sternum and lung, and the other region is on the contralateral side but comprises the same dimensions without including the right ventricle (heart ROI/ CL ROI) [21]. An H/CL ratio ≥1.5 at 1 h after administration of the bone tracer, in association with a Perugini grade ≥2, has a sensitivity of 97% and a specificity of 100% in distinguishing ATTR amyloidosis from the AL form [22]. An H/CL index ≥1.6 has been associated with poor outcome [11].

## 3. Clinical Application of Cardiac Scintigraphy with Bone Tracers

Cardiac scintigraphy with bone tracers has proven to be a crucial technique to allow non-invasive diagnosis of ATTR CA in about 70% of cases [23] as recommended by the position statement of the European Society of Cardiology [24] and serves as a useful tool in differentiating CA from other diseases that cause increased LV thickness [7].

In the presence of a strong clinical, echocardiographic or cardiac MRI suspicion of CA and after ruling out the possible presence of a monoclonal component by serum and urinary immunofixation and serum light chain assay, a cardiac scintigraphy with bone tracers demonstrating a Perugini grade 2 or 3 myocardial uptake confirms the diagnosis of ATTR-CA with an accuracy and specificity of 99% and 100%, respectively [7]. Cardiac or extracardiac histological diagnosis is mandatory in all cases with evidence of monoclonal proteins in serum and/or urine. Initially, Gillmore’s protocol [7] was included and partially revised in the 2021 position statement of the European Society of Cardiology (ESC) Working Group on Myocardial and Pericardial Diseases on Diagnosis and Treatment of Cardiac Amyloidosis [24]. Recently, the ESC guidelines on the management of Cardiomyopathies [25] have been published; regarding cardiac amyloidosis, they substantially refer to the position statement mentioned above. Indeed, in these new guidelines on cardiomyopathies, the typical echocardiographic/cardiac magnetic resonance findings associated with a Perugini grade 2–3 obtained by planar scintigraphy and SPECT with bone tracers (^99m^Tc-PYP, ^99m^Tc-DPD and ^99m^Tc-HMDP) are required for the non-invasive diagnosis of cardiac amyloidosis. In addition to these criteria, the exclusion of a monoclonal component from blood and urine is mandatory. The concept remains essentially unchanged except that more weight is rightly given to tomographic scintigraphy, which the authors of these guidelines wrote “should be considered to reduce the number of misclassifications.” This guidance is also based on the article by Asif et al. [26] Indeed, single-photon emission computed tomography (SPECT) should always be performed following the acquisition of planar imaging, ideally with a hybrid SPECT/CT technique to increase specificity. SPECT allows three-dimensional images to be obtained to better understand the location of the bone tracer accumulation; planar images are no longer recommended in isolation for the work up of patients with suspected CA. For example, SPECT is useful to differentiate myocardial uptake from the persistence of the bone tracer in the ventricular cavity (“blood pool”) or from uptake of thoracic bone structures overlapping the heart (i.e., rib fracture, metastatic bone lesion) [21,27,28]. In addition, SPECT allows focal myocardial uptake to be identified and helps in cases of a falsely negative H/CL index due to patients with previous infarction and thus reduced vital myocardium.

However, there are further scenarios that must be considered to avoid inappropriate applications of scintigraphy or incorrect interpretations with the risk of important clinical implications, such as fatal misdiagnosis or inappropriate utilization of financial and biological resources [29,30].

The mere presence of uptake on bone tracer scintigraphy, which should always be considered an abnormal finding, is not sufficient in isolation for establishing the diagnosis of ATTR-CA as patients with AL CA and apolipoprotein A-I CA may also present with myocardial uptake on cardiac scintigraphy. Although anecdotical, cardiotoxicity from hydroxychloroquine has been associated with myocardial uptake of tracer on scintigraphy with 99mTc-PYP [31]. It is more common, however, to encounter an uptake on bone tracer scintigraphy in the case of rib and sternal fractures, valvular calcifications, cardiac or extra-cardiac masses, recent myocardial infarction, and advanced chronic renal failure/dialytic phase.

The absence of myocardial uptake (i.e., Perugini grade 0) does not always exclude the diagnosis. In patients with suspected CA based on characteristic non-invasive cardiac imaging, the absence of myocardial uptake is still consistent with the low sensitivity of cardiac scintigraphy in presence of rare TTR mutations (i.e., Phe64Leu, Val30Met, Ser77Tyr) [32], non-ATTR forms of amyloidosis [33,34], or AL amyloidosis. Specifically, there are others several scenarios in which a negative result can be observed on cardiac scintigraphy: in very early stages of cardiac amyloidosis, its accumulation may be difficult to detect even with bone tracers; in the presence of a large area of necrosis resulting from a previous infarction, bone tracer accumulation may not occur; if images are acquired too late or too early in the course of radiotracer administration, a false negative may be obtained. If one of these cases is suspected, further tests should be considered to assess for CA.

## 4. Future Directions

### 4.1. Why Do Bone Tracers Work in Cardiac Amyloidosis?

The precise mechanism by which bone tracers’ function in amyloidosis is still unknown, although various hypotheses have been generated over the years. For example, as previously stated, certain studies support the possibility that bone tracers may bind microcalcifications that are more represented in ATTR CA than AL. However, histologically, only a percentage of patients show significant calcifications that are more represented at bone level compared with the cardiac level in each case, yet the uptake of bone tracers remains greater at the cardiac level. One study demonstrated that cardiac scintigraphy with bone tracers was very sensitive in diagnosing the disease in patients with a greater presence of type A amyloid fibrils; however, in patients with only type B fibrils, scintigraphy did not detect them [17]. Therefore, calcifications are probably not the only mechanism at play, not least because we are not sure of the ultimate ligand of the bone tracer, which could therefore be indicative of something else (amyloid, extracellular matrix, other factors).

### 4.2. Initial Cardiac Involvement

Using Gillmore’s algorithm, it is possible to diagnose phenotypically expressed disease, meaning that there must be clinical-instrumental suspicion to initiate the non-invasive diagnostic work-up. However, subjects with cardiac uptake on scintigraphy but asymptomatic and without a clinical context suggestive of amyloidosis are excluded from Gillmore’s algorithm. This suggests that there is a difference between simple cardiac amyloid deposition and amyloid deposition in the context of a pathology, that is, amyloidotic cardiomyopathy (AC).

### 4.3. Negative Scintigraphy but Suspicious Phenotype, When to Perform Further Examinations?

Similarly, there are subjects with echocardiographic and cardiac MRI criteria suggestive of cardiac amyloidosis with, however, reduced or no uptake on cardiac scintigraphy (e.g., Perugini grade 0 or 1). In this case, the instrumental/scintigraphic discrepancy raises the suspicion of rare amyloidosis subtypes, such as AL amyloidosis, ApoAI and ApoAIV amyloidosis, and certain ATTRvs (Val30Met, Phe64Leu, Ser77Tyr).

In a study by Musumeci et al. [35], it was observed that cardiac scintigraphy with bone tracers was negative in 89% of patients with Phe64Leu ATTRv and had a reduced uptake in Val30Met ATTRv. Furthermore, Ser77Tyr also showed reduced uptake on scintigraphy with ^99m^Tc-DPD despite typical echocardiographic and cardiac MRI features [9,36]. Clearly these findings suggest that the various ATTR subtypes may exhibit different pathogeneses that are linked to different biological mechanisms [37].

In these cases, SPECT-TC is very useful because it allows focal/regional radiotracer uptake to be recognized, as cardiac uptake often begins in the basal areas of the inferior interventricular septum and then extends in a baso-apical and septal-lateral direction.

### 4.4. Is Scintigraphy Useful in Asymptomatic TTR Gene Variant Carriers?

In cases of ATTRv in which an asymptomatic subject without echocardiographic or cardiac MRI criteria suggestive of amyloidosis is a carrier of a known mutation, scintigraphy could be used as a screening method since it detects cardiac involvement early even when the MRI is normal. Even a low-grade cardiac uptake (e.g., Perugini grade 1) configures cardiac involvement at an early stage [38].

### 4.5. Is Cardiac Scintigraphy with Bone Tracers Only Useful for Diagnosis? Is It Also Useful for Tracking Progression of Heart Disease and Assessing Prognosis?

Cardiac scintigraphy with bone tracers also seems to provide rough information for prognosis: patients with Perugini grade 2 or 3 myocardial uptake in ATTR amyloidosis have a poorer outcome compared to those presenting with Perugini grade 1 myocardial uptake [9,39,40]. However, the use of different bone tracers in different countries and different acquisition times limits the possibility of comparison. In addition, scintigraphy cannot be the method of choice in assessing disease progression or evaluating the response to specific therapies since it still creates biological damage and especially due to ignorance of the principles guiding cardiac uptake [41].

Rather, the use of SPECT improves diagnostic accuracy and can help in monitoring disease progression and response to specific therapy. In a recent work by Genovesi et al., it was shown that late cardiac uptake of [18F]-florbetaben by PET/CT may be able to discriminate ATTR amyloidosis from AL, possibly paving the way for non-invasive diagnosis even in AL amyloidosis. [42] Further studies are needed given the significant variability of findings in individual patients [43]. 

Recently, Porcari et al. proposed that right ventricular uptake on cardiac scintigraphy may be associated with an increased risk of all-cause mortality among patients with ATTR-CA [44]. Although the reason is unclear, the authors speculate that biventricular uptake (left and right ventricle) may reflect a more advanced disease and a more advanced cardiac amyloid infiltration compared to isolated LV uptake. If confirmed in further dedicated research, characterization of the presence and extent of RV uptake might become the first solid nuclear imaging parameter associated with overall survival among patients with either wild-type or variant ATTR-CA.

## 5. Conclusions

The development of a non-biopsy algorithm for confirmation of ATTR-CA has deeply transformed the diagnosis approach for a patient with suspected disease. Cardiac scintigraphy with bone tracer has entered the clinical arena and is now widely used worldwide as diagnostic tool coupled with exclusion of monoclonal protein in serum and urine. However, the mechanisms of action and the binding site of bone tracers in the amyloid heart still represent a great mystery that is crucial to understand to advance the clinical application of this technique for prognostication and monitoring treatment response.

## Figures and Tables

**Table 1:** Cardiac evaluation methods in scintigraphy with bone tracers.

Score	Grade
Perugini	Grade 0: no cardiac uptake; Grade 1: mild cardiac uptake, less than bone uptake; Grade 2: moderate cardiac uptake accompanied by attenuated bone uptake; Grade 3: strong cardiac uptake with mild/absent bone uptake
Perugini modified by Dorbala	Grade 0: no myocardial uptake and normal bone uptake Grade 1: myocardial uptake less than rib uptake Grade 2: myocardial uptake equal to rib uptake Grade 3: myocardial uptake greater than rib uptake with mild/absent rib uptake
H/CL (heart to controlateral lung)	H/CL ratio ≥ 1.5 at one hour

## Data Availability

Not applicable.
